# First Molecular Evidence for Underestimated Biodiversity of *Rhachotropis* (Crustacea, Amphipoda), with Description of a New Species

**DOI:** 10.1371/journal.pone.0032365

**Published:** 2012-03-01

**Authors:** Anne-Nina Lörz, Katrin Linse, Peter J. Smith, Dirk Steinke

**Affiliations:** 1 National Institute of Water and Atmospheric Research, Kilbirnie, Wellington, New Zealand; 2 British Antarctic Survey, Cambridge, United Kingdom; 3 Museum Victoria, Melbourne, Victoria, Australia; 4 Biodiversity Institute of Ontario, University of Guelph, Guelph, Ontario, Canada; Brigham Young University, United States

## Abstract

The crustacean genus *Rhachotropis* has a worldwide distribution and amongst the largest bathymetric range known from any amphipod genus. DNA barcoding of new material from around New Zealand and the Ross Sea indicated depth-related biogeographic patterns. New Zealand *Rhachotropis* do not form a monophyletic clade. Species from bathyal depths on the Chatham Rise, east of New Zealand, show lower sequence divergence to bathyal species from California and the Arctic than to abyssal New Zealand species. Species sampled in the Kermadec Trench, north of New Zealand below 5000 m, seem to be more closely related to Ross Sea abyssal species than to the New Zealand shelf species. The worldwide geographic and bathymetric distribution for all *Rhachotropis* species is presented here. Depth may have a greater influence on phylogeny than geographic distance.

Molecular and morphological investigations of *Rhachotropis* specimens from the Chatham Rise, New Zealand revealed a species new to science which is described in detail, including scanning electron microscopy. This increases the number of described species of *Rhachotropis* to 60 worldwide.

## Introduction

**Figure 1:**
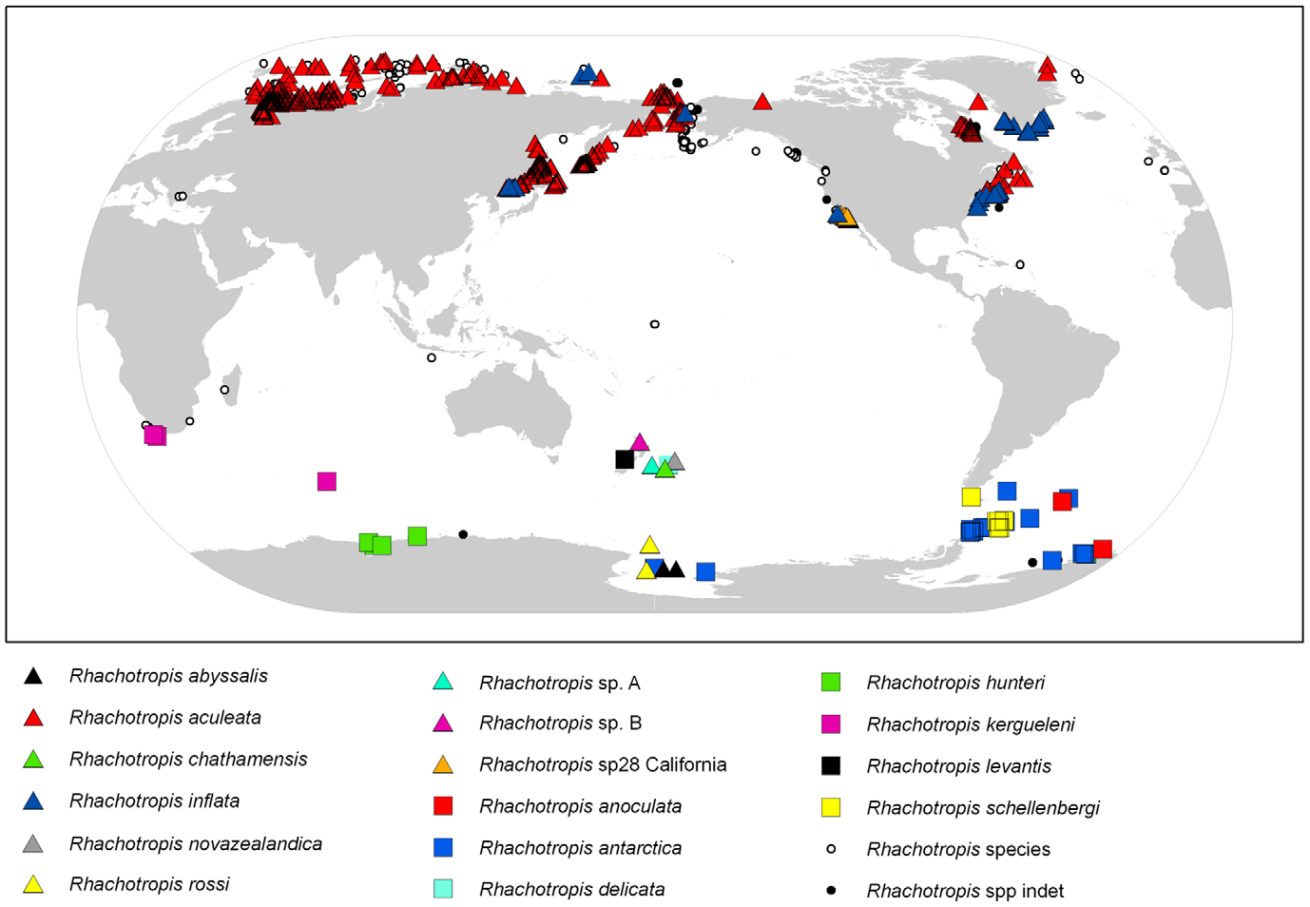
Global distribution map of the genus *Rhachotropis* with the species represented in the molecular part of this paper in triangles, the Southern Ocean species in squares and the remaining species (∼40), including unidentified ones in small circles.

The amphipod genus *Rhachotropis* (Eusiridae) contains 59 known species with a worldwide distribution (Fig. 1), [1]. *Rhachotropis* species appear to have a patchy distribution although some species are locally very abundant [1], [2], especially in benthic slope communities [3]. Species in general have a relatively high swimming capacity, indicative of a partial pelagic lifestyle [3]. Phylogenetic analyses based on morphological characters have been unsatisfying or not possible. The numerical analysis of 20 morphological characters and corresponding character states by Bousefield & Hendrycks [4] focused on gross external morphology rather than mouthparts and reproductive features that may actually prove more significant phylogenetically as the authors suggested. Even though *Rhachotropis* show an impressive horizontal and vertical distribution, the genus has not been studied with molecular phylogenetic tools. This is a first preliminary analysis of the mitochondrial **cytochrome oxidase** c subunit **1** (COI) sequences of *Rhachotropis* specimens collected from bathyal and abyssal depths around New Zealand and in the Ross Sea.

This paper describes one new species collected on the Chatham Rise, east of New Zealand, and increases the number of known and described *Rhachotropis* species to 60, and the New Zealand *Rhachotropis* to four species. At least two further species from New Zealand waters appear to be new, but in too poor condition as to be formally described.

## Results

### COI

**Figure 2:**
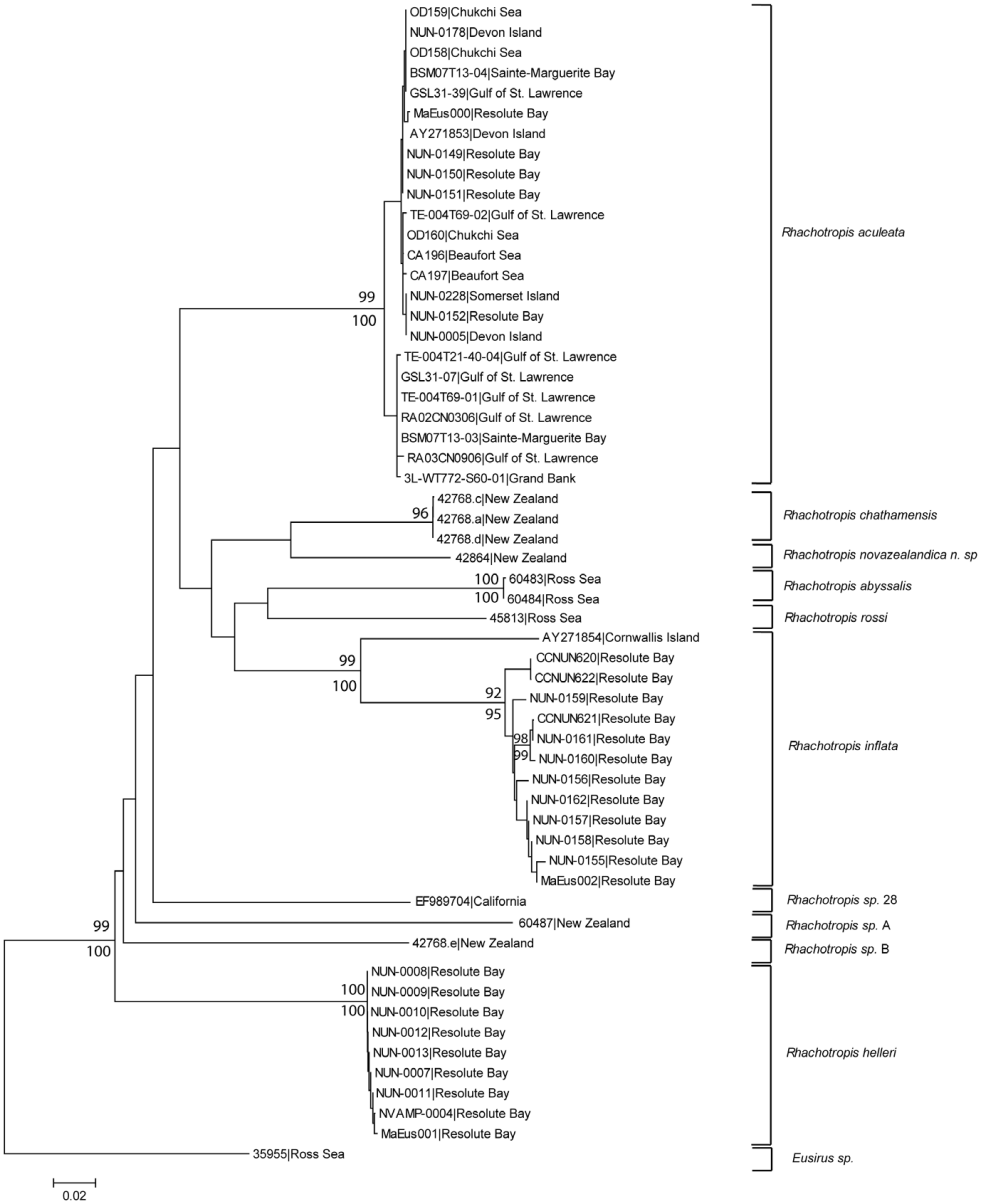
Relationships of COI sequences from *Rhachotropis* specimens. BOLD Accession Numbers are given for each specimen along with locations. Numbers at nodes are ML bootstrap percentages (>80%) after 1000 replications, and Bayesian inference posterior probability values (>0.90); scale bar represents an interval of the TIM+I+G model. The tree topology represents the 50% majority rule consensus of all Bayesian trees and has been rooted with the Antarctic *Eusirus*.

Relationships for nine *Rhachotropis* specimens from New Zealand and the Ross Sea are shown in Fig. 2 and represent the topology inferred by both analyses. The trees were rooted with the Antarctic outgroup *Eusirus* sp., and include additional close matches for northern hemisphere *Rhachotropis* COI sequences held in Genbank: *R. inflata*, *R. aculeata*, *R. inflata*, *R. helleri*, *and a putative new species from California R*. sp 28 (Table 1). The DNA barcodes revealed six well supported clades of *Rhachotropis* specimens from New Zealand and the Ross Sea with a further four clades for the northern hemisphere species (Fig. 2). Three specimens from the Chatham Rise, New Zealand, had identical COI sequences and were described as *R. chathamensis* Lörz, 2010. Two specimens from the Ross Sea had identical COI sequences and belong to *R. abyssalis* Lörz, 2010. A further three specimens from New Zealand had three unique COI sequences; one specimen which is described in this paper as *R. novazealandica*, Lörz, 2012 (Fig. 2), while the other two specimens remain undescribed: R. sp. A and R. sp. B (Fig. 2). A fourth single specimen from the Ross Sea with a unique sequence was described as *R. rossi* Lörz, 2010 (Fig. 2).

**Table 1:** *Rhachotropis* and outgroup accession numbers in BOLD, GenBank and station data.

Species	BOLD	Sample ID	GenBank Acc#	Area	Lat	Lon	Depth
*Rhachotropis abyssalis*	AMPNZ095-09	60483	GU804296	Ross Sea	−76.19	176.30	447
*Rhachotropis abyssalis*	AMPNZ094-09	60484	GU80484	Ross Sea	−76.19	176.30	447
*Rhachotropis aculeata*	WWGSL070-08	GSL31-39	FJ581879	St. Lawrence Gulf	48.15	−63.54	
*Rhachotropis aculeata*	WW865-08	GSL31-07	FJ581880	St. Lawrence Gulf	47.90	−65.35	
*Rhachotropis aculeata*	WWGSL098-08	TE-004T21-40-04	FJ581881	St. Lawrence Gulf	48.39	−59.55	150
*Rhachotropis aculeata*	WW851-08	TE-004T69-02	FJ581882	St. Lawrence Gulf	50.82	−58.59	233
*Rhachotropis aculeata*	WW850-08	TE-004T69-01	FJ581883	St. Lawrence Gulf	50.82	−58.59	233
*Rhachotropis aculeata*	WW105-07	RA03CN0906	FJ581884	St. Lawrence Gulf	49.92	−64.62	
*Rhachotropis aculeata*	WW129-07	RA02CN0306	FJ581885	St. Lawrence Gulf	51.14	−58.05	
*Rhachotropis aculeata*	WW459-08	BSM07T13-04	FJ581886	Cote-Nord	50.25	−66.70	
*Rhachotropis aculeata*	WW458-08	BSM07T13-03	FJ581887	Cote-Nord	50.25	−66.70	
*Rhachotropis aculeata*	BENTH312-08	OD158	JQ412470	Chukchi Sea	70.00	−168.40	45
*Rhachotropis aculeata*	BENTH313-08	OD159	JQ412471	Chukchi Sea	70.00	−168.40	45
*Rhachotropis aculeata*	BENTH314-08	OD160	JQ412469	Chukchi Sea	70.00	−168.40	45
*Rhachotropis aculeata*	WW402-08	3L-WT772-S60-01	JQ412480	Grand Bank	46.61	−49.24	74
*Rhachotropis aculeata*	CCNUN228-07	NUN-0228	JQ412476	Somerset Island	72.77	−93.36	
*Rhachotropis aculeata*	CCNUN149-07	NUN-0149	JQ412465	Resolute	74.68	−94.86	
*Rhachotropis aculeata*	CCNUN150-07	NUN-0150	JQ412468	Resolute	74.68	−94.86	
*Rhachotropis aculeata*	CCNUN151-07	NUN-0151	JQ412467	Resolute	74.68	−94.86	
*Rhachotropis aculeata*	CCNUN152-07	NUN-0152	JQ412466	Resolute	74.68	−94.86	
*Rhachotropis aculeata*	CCNUN178-07	NUN-0178	JQ412475	Devon Island	74.67	−91.70	
*Rhachotropis aculeata*	CCNUN005-07	NUN-0005	JQ412473	Devon Island	75.76	−88.12	
*Rhachotropis aculeata*	RBGC036-03	MaEus000	DQ889127	Resolute			
*Rhachotropis aculeata*	WW023-07	CA196	JQ412474	Beaufort Sea	70.90	−128.90	
*Rhachotropis aculeata*	WW024-07	CA197	JQ412472	Beaufort Sea	70.90	−128.90	
*Rhachotropis aculeata*	GBCMA0080-06	AY271853	AY271853	Resolute			
*Rhachotropis chathamensis*	AMPNZ101-09	42768.d	GU804298	New Zealand	−43.80	175.32	418
*Rhachotropis chathamensis*	AMPNZ100-09	42768.c	GU804299	New Zealand	−43.80	175.32	418
*Rhachotropis chathamensis*	AMPNZ098-09	42768.a	GU804300	New Zealand	−43.80	175.32	418
*Rhachotropis helleri*	CCNUN449-08	NVAMP-0004	JQ412483	Resolute	75.08	−94.86	
*Rhachotropis helleri*	CCNUN007-07	NUN-0007	JQ412484	Resolute	74.68	−94.86	
*Rhachotropis helleri*	CCNUN008-07	NUN-0008	JQ412482	Resolute	74.68	−94.86	
*Rhachotropis helleri*	CCNUN009-07	NUN-0009	JQ412481	Resolute	74.68	−94.86	
*Rhachotropis helleri*	CCNUN010-07	NUN-0010	JQ412477	Resolute	74.68	−94.86	
*Rhachotropis helleri*	CCNUN011-07	NUN-0011	JQ412480	Resolute	74.68	−94.86	
*Rhachotropis helleri*	CCNUN012-07	NUN-0012	JQ412485	Resolute	74.68	−94.86	
*Rhachotropis helleri*	CCNUN013-07	NUN-0013	JQ412479	Resolute	74.68	−94.86	
*Rhachotropis helleri*	RBGC037-03	MaEus001	JQ412478	Resolute			
*Rhachotropis inflata*	CCNUN620-08	CCNUN620	JQ412491	Resolute	75.08	−94.86	
*Rhachotropis inflata*	CCNUN621-08	CCNUN621	JQ412492	Resolute	75.08	−94.86	
*Rhachotropis inflata*	CCNUN622-08	CCNUN622	JQ412493	Resolute	75.08	−94.86	
*Rhachotropis inflata*	CCNUN334-07	NUN-0334	JQ412487	Igloolik	69.37	−81.79	
*Rhachotropis inflata*	CCNUN154-07	NUN-0154	JQ412489	Resolute	74.68	−94.86	
*Rhachotropis inflata*	CCNUN155-07	NUN-0155	JQ412498	Resolute	74.68	−94.86	
*Rhachotropis inflata*	CCNUN156-07	NUN-0156	JQ412488	Resolute	74.68	−94.86	
*Rhachotropis inflata*	CCNUN157-07	NUN-0157	JQ412497	Resolute	74.68	−94.86	
*Rhachotropis inflata*	CCNUN158-07	NUN-0158	JQ412499	Resolute	74.68	−94.86	
*Rhachotropis inflata*	CCNUN159-07	NUN-0159	JQ412495	Resolute	74.68	−94.86	
*Rhachotropis inflata*	CCNUN160-07	NUN-0160	JQ412494	Resolute	74.68	−94.86	
*Rhachotropis inflata*	CCNUN161-07	NUN-0161	JQ412490	Resolute	74.68	−94.86	
*Rhachotropis inflata*	CCNUN162-07	NUN-0162	JQ412496	Resolute	74.68	−94.86	
*Rhachotropis inflata*	RBGC038-03	MaEus002	JQ412486	Resolute			
*Rhachotropis inflata*	GBCMA0081-06	AY271854	AY271854	Resolute			
*Rhachotropis novazealandica* n. sp.	AMPNZ128-09	42864	GU804309	New Zealand	−44.13	174.85	520
*Rhachotropis rossi*	ANZR470-08	45813	JF498593	Ross Sea	−76.59	176.83	369
*Rhachotropis sp.* 28	GBCMA1154-08	EF989704	EF989704	California	36.33	122.90	300–700
*Rhachotropis sp.* A	AMPNZ184-10	60487	JF498594	New Zealand	−36.52	179.20	5173
*Rhachotropis sp*. B	AMPNZ102-09	42768.e	HM372956	New Zealand	−43.80	175.32	418
*Eusirus sp.* (outgroup)	ANZR028-08	35955	JQ412464	Ross Sea	−72.08	175.55	1620

Sequence divergence was zero within the *R. chathamensis* and *R. abyssalis* clades, and low within the 24 specimens of *R. aculeata* (0.0089), 9 specimens of *R. helleri* (0.0003), and 13 specimen of *R. inflata* (0.037). A single specimen identified as *Rhachotropis inflata* (Cornwallis Island) is separated distinctly from the remaining clade (separated by 3% sequence divergence). And one tentative species, *R*. sp 28 from California, is represented by one sequence retrieved from GenBank. Inter-clade sequence divergences ranged from 0.143–0.370 with an overall average divergence 0.284. The lowest divergence (0.143, Table 2) was between *R. novazealandica* spec. nov. from New Zealand and R. sp. 28 from California, while the greatest divergence was between the two putative species *R.* sp. A and *R. sp. B* (0.370, Table 2) from New Zealand.

**Table 2:** Nucleotide distances (TIM+I+G) within and between species/clades of *Rhachotropis*.

	N	within	*R. aculeata*	*R. inflata*	*R. helleri*	*R. abyssalis*	*R. chathamensis*	*R. sp.* 28	*R. rossi*	*R. zealandica*	*R. sp.* A	*R. sp.* B	Outgroup
*R. aculeata*	24	0.00887											
*R. inflata*	13	0.03672	0.27756										
*R. helleri*	9	0.00035	0.26149	0.27599									
*R. abyssalis*	2	0	0.25909	0.26891	0.29778								
*R. chathamensis*	3	0	0.22547	0.25206	0.19702	0.28893							
*R. sp.* 28	1	n/a	0.24149	0.30575	0.27101	0.31554	0.2622						
*R. rossi*	1	n/a	0.2502	0.28218	0.28517	0.28076	0.2164	0.26698					
*R. novazealandica*	1	n/a	0.23667	0.26022	0.24123	0.27004	0.2435	0.14342	0.24142				
*R. sp.* A	1	n/a	0.30815	0.32293	0.31698	0.30095	0.3544	0.31781	0.31284	0.3328			
*R. sp.* B	1	n/a	0.26806	0.30257	0.25556	0.29027	0.3214	0.30855	0.30804	0.315	0.3699		
Outgroup	1	n/a	0.30998	0.30515	0.29003	0.30271	0.3229	0.28589	0.3078	0.3151	0.2916	0.365	

N = number of specimens.

Morphological investigation revealed a species new to science which is described herein. Even though only a single damaged specimen exists, the COI sequence and detailed morphological descriptions will allow corroboration by future collections.

### Systematics

Order AMPHIPODA Latreille, 1816

Suborder GAMMARIDEA Latreille, 1802

Family EUSIRIDAE Stebbing, 1888

Genus *Rhachotropis* S.I. Smith, 1883

*Rhachotropis novazealandica* spec. nov.

**Figure 3:**
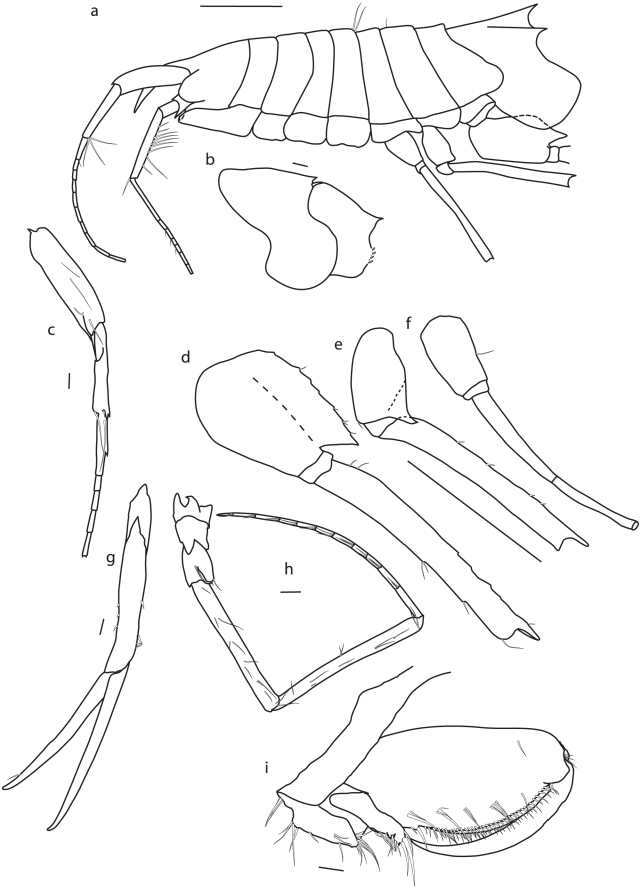
*Rhachotropis novazealandica* spec. nov., female holotype NIWA 42864. a) habitus lateral, b) epimeral plates 2 and 3, c) antenna 1, d) pereopod 7, e) pereopod 6, f) pereopod 5, g) uropod 1, h) antenna 2, i) gnathopod 1 Scalebars:a,d,e,f = 1 mm; b = 200 µm; c,g,h,i = 100 µm.

**Figure 4:**
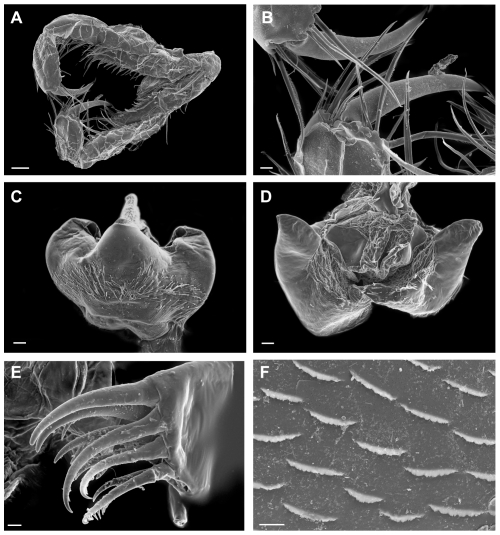
*Rhachotropis novazealandica* spec. nov., female holotype NIWA 42864. A) maxilliped, B) dactyli of maxillipedal palp, C) labrum, D) hypopharynx, E) maxilla 1 outer lobe, F) surface on epimal plate 2. Scalebars: A = 100 µm, B, C, D = 20 µm; E, F = 10 µm.

**Figure 5:**
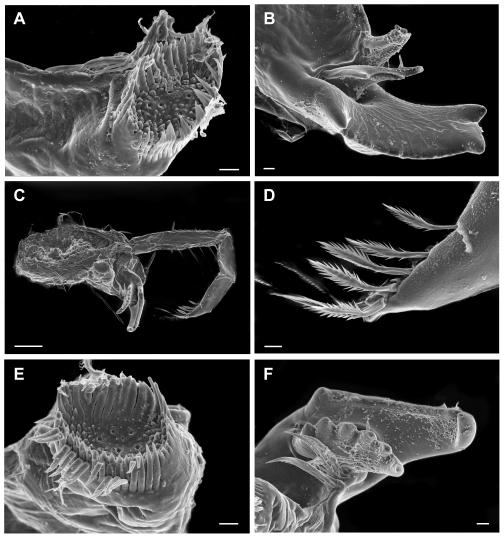
Mandible of *Rhachotropis novazealandica* spec. nov., female holotype NIWA 42864. A) molar, B) incisor and lacina mobilis right mandible, C) left mandible, D) mandibular palp terminal end, E) molar, F) incisor and lacina mobilis, left mandible. Scalebars: A, B, D, E, F = 10 µm, C = 100 µm.

**Figure 6:**
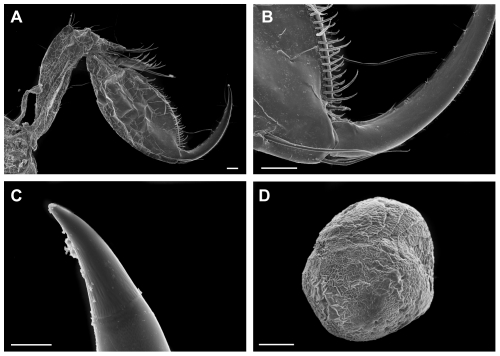
*Rhachotropis novazealandica* spec. nov., female holotype NIWA 42864. A) Gnathopod 1 v 2, B) palm of gnathopod 1 v 2, C) tip of dactylus, D) egg. Scalebars: A, B, D = 100 µm, C = 10 µm.

**Figure 7:**
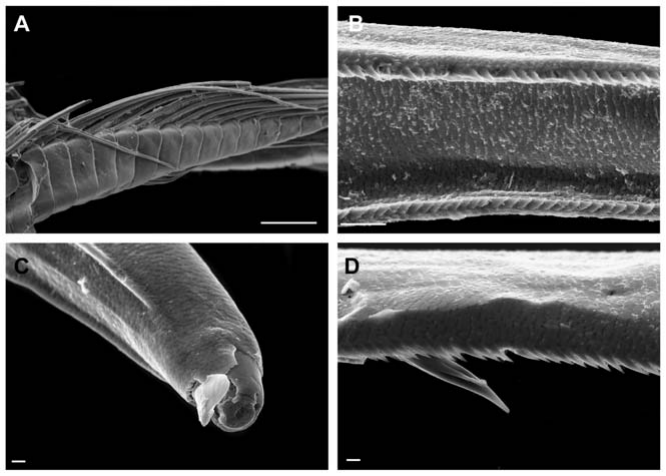
*Rhachotropis novazealandica* spec. nov., female holotype NIWA 42864. A) rami of pleopod 1, B) mid rami of uropod 1, C) tip of rami uropod 1, D) setation on peduncle margin of uropod 1. Scalebars: A = 100 µm, B = 10 µm, C, D = 2 µm.

Lörz, 2012

(Figs. 3, 4, 5, 6, 7)

#### Material examined

Holotype: NIWA 42864, female, 17 mm TAN0705/12, 13 Apr 2007, Box corer at 520 m, 44* 7.57 S, 174* 50.74E, R.V. Tangaroa, Collector: Ocean Survey 20/20 Chatham Rise, New Zealand.

#### Etymology

*Rhachotropis novazealandica* spec. nov. is named after New Zealand where the species was collected.

#### Diagnosis

Body delicate. Rostrum longer than head. Eyes absent. Head twice as long as pereonite 1, lateral lobes produced. Pereonites smooth. All pleonites bearing dorsal processes, pleonite 1 also bearing dorsolateral processes.

#### Description

Antenna 1 second article of peduncle with several plumose setae, article 2 slightly shorter than article 1, twice as long as article 3; flagellum broken after 10th-articulate. Antenna 2 peduncle article 3 longer than article 4, several plumose setae on third article; flagellum broken after 6^th^ article.

Mandible with smooth incisor process well developed, lacinia mobilis denticulate, molar process conical. Left and right molars have several pores in the middle. The tip of the left mandibular palp bears six plumose setae. Maxilla 1 inner plate bearing 1 subterminal seta, outer plate with 9 denticulate spines. Maxilliped outer plate 2.5 times as long as inner plate, reaching half of article 2 of maxillipedal palp; inner margins of palp, outer plate and terminal end of inner plate setose. Labrum entire, smooth and broadly rounded. Hypopharynx setose, outer lobes with broad gap.

Gnathopod 1 coxa 1 produced, reaching to end of head, coxa 2, 3 and 4 subquadrate. Gnathopods similar in shape, subchelate. Gnathopod 1 slightly smaller than gnathopod 2, basis bearing several small spines at anterior side; merus with long setae at posteroventral corner; carpus lobe extending width of propodus, spines at terminal end of lobe; propodus widened, oval; dactylus slender, reaching end of palm.

Pereopod 5 basis small, narrow; merus longer than carpus. Pereopod 6 basis larger than of pereopod 5, posteroventral angle produced. Pereopod 7 basis widened, posterior margin serrate, posteroventral angle strongly produced; merus posteroventral angle produced.

Uropod 1 rami same length.

#### Remarks

*Rhachotropis novazealandica* spec. nov. differs from the other four *Rhachotropis* species from New Zealand (*R. chathamensis* Lörz 2010; *R. delicata* Lörz 2010; *R. levantis* Barnard 1961 and *R. spec* Dahl, 1959) by the combination of following characters: rounded coxa 1 (vs R. *chathamensis*), coxa 2 smaller than coxa 3 (vs *R. chathamensis*), coxa 3 and 4 ventral margin slightly bilobed (vs straight *R. chathamensis*), second segment of maxillipedal palp not broadend (vs R. spec Dahl, 1959) gnathopod 2 propodus extension exceeding article (as *R. delicata* vs *R. levantis*), uropod 1 rami same length (as *R. delicata*, vs *R. levantis*), gnathopod 1 and 2 dactylus as long as palm, basis pereopod 6 and 7 strong dorsolateral projection (vs *R. delicata*).

#### Distribution

New Zealand, Chatham Rise, 520 m.

## Discussion

This is the first molecular study of *Rhachotropis* and has revealed a high level of diversity among specimens from the northern and southern hemispheres. The historic *Rhachotropis* collections, including the type material for most the species, were preserved in formalin or other DNA degrading media and are therefore not suitable for routine molecular investigations. Some fragile *Rhachotropis* specimens collected on recent expeditions were damaged and unsuitable for detailed morphological descriptions, but were fixed in ethanol to enable molecular studies. This study continues the integrative approach of DNA barcoding and classic taxonomy.

Most barcode projects aim to develop open-access libraries derived from referenced (vouchered) specimens that will improve understanding of biodiversity, highlight cryptic species, and provide rapid tools for identification of a wide range of species [5], [6], [7]. While barcoding has its limitations, especially the discrimination of recently diverged species that underwent introgressive hybridisation, the COI barcode region has been shown to be appropriate for discrimination between closely related species across diverse animal phyla [7]–[10]. Barcoding can highlight potentially cryptic species that appear in discrete clades with high sequence divergences as in the *Rhachotropis* case here. High intra-specific divergences indicate that additional data are required to distinguish potential new species from known species. The barcode databases, once established can be applied to the DNA identification of specimens where traditional morphological methods are inappropriate such as stomach contents in fishes [11], [12], fish fillets [13], [14] and environmental barcoding for biomonitoring [15].

Although there are ongoing discussions about the level of intra- and inter-specific divergences in amphipods and the concept of species to be used [16], [17] molecular species recognition is mostly based on the barcode “gap” between intra- and interspecific variations, with high inter- and low intra-clade sequence divergences indicative of cryptic species. Based on the barcode gap and consistent morphological differences, Lörz et al. [18] described and redescribed species of Antarctic Amphipoda and suggested that benthic species of Amphipoda do not occur circum Antarctic.

The inter- and intra-specific divergences of the *Rhachotropis* clades are in the same order of magnitude as for other deep sea Amphipoda (e.g. [10], [18], [19]). Interspecific uncorrected COI sequence distances in the Antarctic Iphimediidae varied from 7.9% (*Echiniphimedia scotti* to *E. hodgsoni*) to 29.5% (*Iphimediella cyclogena* to *I. georgei*) [18]. The deepwater Antarctic *Rhachotropis* species from the Admiralty seamount and Scott Island, to the north of the Ross Sea were in the same range, 28%.

Within the Epimeriidae sequence divergence varied from 8.5% (*E. schiaparelli* to *E. macrodonta*) to 26.15% (*E. horsti* to *E. annabellae*) [18]. Sequences of species from New Zealand's seamounts, *Epimeria horsti* and *E. bruuni* were more similar to each other than to any of the remaining Antarctic *Epimeria* species, but the distance between them was high with nearly 20%. The Antarctic *Epimera* species formed a monophyletic clade [18] while this study found the New Zealand *Rhachotrois* not to be monophyletic with the largest genetic distance of 37% between species..

**Figure 8:**
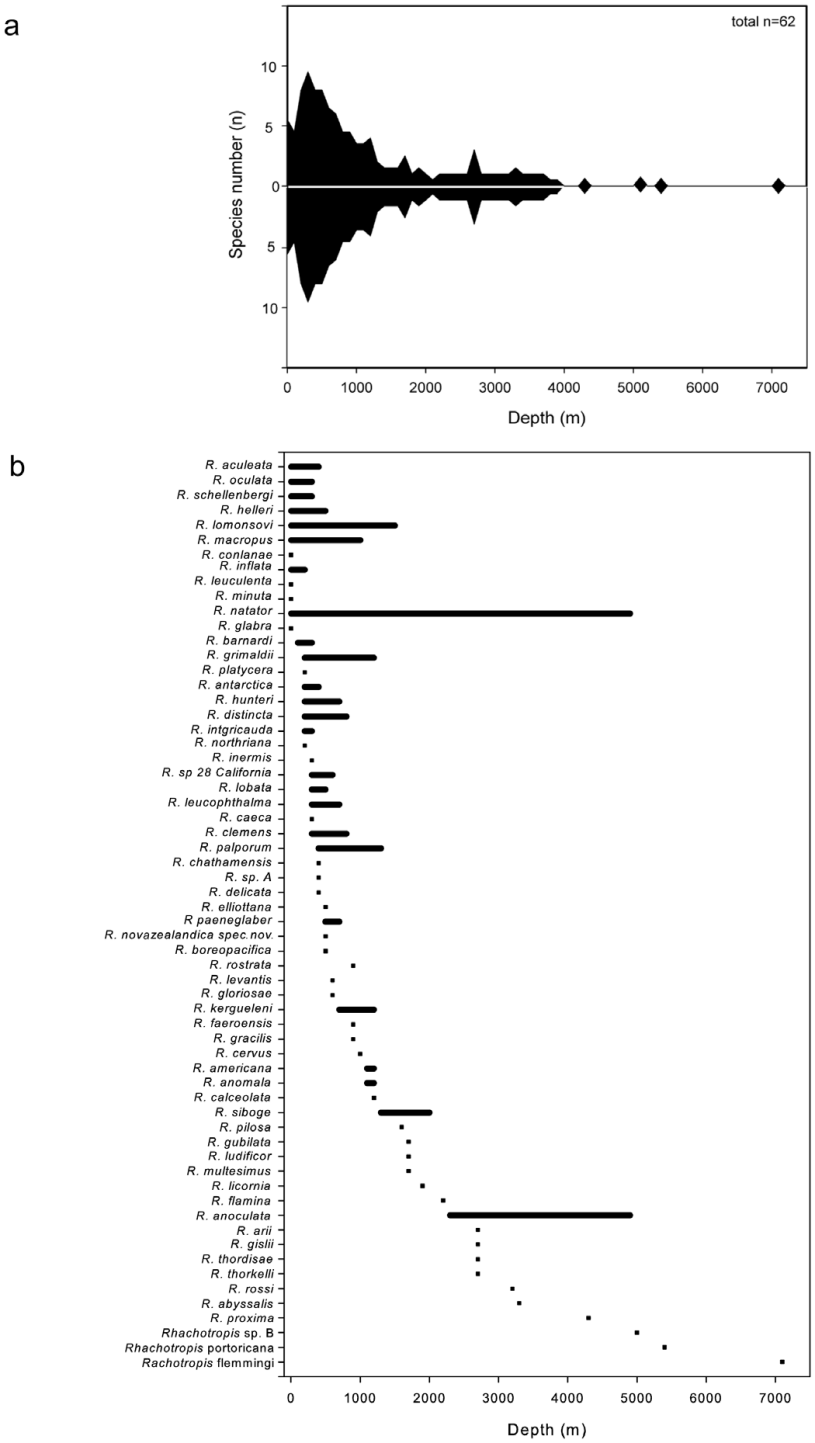
a) Depth distribution of *Rhachotropis* species showing that the genus spans from the shallow to the abyss; b) the depth range of the 59 named plus the 3 spp from this study (A, B and sp. 28 California) *Rhachotropis* species.

*Rhachotropis* specimens are found in all major oceans of the world: Arctic, Atlantic Ocean, Mediterranean Sea, Carribean Sea, Indian Ocean, Pacific Ocean and the Southern Ocean (see Fig. 1 map). *Rhachotropis* specimens have been collected in all water depths (see Fig. 8a,b), from the shelf (e.g. [20]) to abyssal and hadal sampling sites (*R. rossi*, *R. abyssalis* Lörz 2010), in trenches (*R. flemmingi* Dahl 1959, Sunda Trench 7160 m; *R. spec A* Kermadec Trench, 7180, Dahl 1959), as well as around hydrothermal vents (e.g. [21]). Specimens used in this study are from three oceans, the Arctic, Southern and Pacific Oceans. Generally more species are currently known from the shelf and upper slope area, however, the observed depth pattern is heavily sample/collection biased and areas with more stations show more species. For example, detailed sampling at one deepwater location (2700 m Iceland Basin) shows four species. Similar results are found for Southern Ocean species in general [22] and in specific groups, such as isopods and gastropods [23].

The worldwide and broad depth distribution makes *Rhachotropis* an ideal model group to test the relationship between shelf and trench faunas or biogeographic “processes” such as sub– or emergence events. Submergence describes the downwards movement/shift of taxa from the shelf/shallow water depth to deeper waters (continental slopes and abyss) while emergence represents the upward movement of taxa from deeper to shallower depth [24] Currently there is insufficient specimen or habitat coverage to provide such comparisons and present a phylogeny of the genus, but this snapshot of *Rhachotropis'* molecular biodiversity provides an indication of what could be found with integrative methods and extensive sampling.

Our preliminary study suggests that the New Zealand *Rhachotropis* fauna is not monophyletic (Fig. 2), with the highest sequence divergence among all *Rhachotropis* specimens found between two species from New Zealand waters, *R.* sp. A from the Kermadec Trench (>5000 m), and *R.* sp. B, sampled from the Chatham Rise, east of New Zealand (418 m). Their position in the tree remains to be inconclusive with no node support. This suggests the use of additional molecular markers in subsequent studies. Based on COI New Zealand bathyal species seem to be closer related to Californian and Arctic shelf species than to New Zealand abyssal species. The New Zealand trench specimen shows a divergence of 30% to the Antarctic abyssal species, sampled below 3000 m at the Admiralty seamount and Scott Island. We therefore hypothesise that depth has a greater influence on the phylogeny of *Rhachotropis* than geography. The Kermadec Trench is one of the coldest trenches in the world [25]. The Deep Western Boundary Current purges Antarctic Bottom Water from the southern entrance into the Kermadec Trench [26], and it appears likely that the New Zealand trench species derive from Antarctica. However, further studies with additional molecular markers are needed to better resolve the tree and to support this hypothesis.

Further specimens from a comprehensive species set, from the shelf to abyssal and hadal depths, and additional genetic markers are required to test sub- or emergence theories. Our preliminary analyses testing DNA divergence against geography (Fig. 1) and depth (Fig. 8a, b), indicate that *Rhachotropis* could be a deep-sea taxon that has undergone several speciation events establishing it at bathyal depths (Emergence) in oceans around the world.

## Materials and Methods

All necessary permits were obtained for the described field studies. Studies in the Ross Sea were undertaken under permit number AMLR07/005/Tangaroa/ZMFR, issued by the New Zealand Government by the Minister of Fisheries Jim Anderton on 19 December 2007 under New Zealand Antarctic Marine Living Resources Act 1981, for the CCAMLR statistical subareas 88.1 and 88.2. Collection of bio samples from the Kermadec Trench expedition (KAH0910) and for the Oceans Survey 2020 Chatham Challenger project (TAN0705) was undertaken under Special Permits (421 and 318) issued by the Ministry of Fisheries pursuant to section 97 (1)(i) and (ii) of the New Zealand Fisheries Act 1996.

### Taxon sampling

*Rhachotropis* amphipods were collected during the Ocean Survey 2020 voyages of RV Tangaroa to the Chatham Rise 2007 (TAN0705) east of New Zealand and to the western Ross Sea 2008 (IPY-CAML, TAN0802), and during the RV Kaharoa voyage HADEEP #6 to the Kermadec Trench north of New Zealand 2009 (KAH0910). Specimens were immediately sorted on deck, often photographed alive on board to record live coloration, fixed in 98% ethanol and later transferred to 70% ethanol.

The amphipod specimens were identified to species level by the first author using identification keys based on morphological characters.

The amphipod specimens including the type material have been registered and curated at the National Institute for Water & Atmospheric Research (NIWA) Invertebrate Collection (NIC) in Wellington, New Zealand.

### DNA extraction and analyses

DNA was extracted from a sub-sample of muscle tissue from nine specimens using an automated Glass Fiber protocol [27]. The 650 bp barcode region of COI was amplified under the following thermal conditions: 1 min at 94°C; 5 cycles of 94°C for 40 s, 45°C for 40 s and 72°C for 1 min, followed by 35 cycles at 94°C for 40 s, 40 s at 51°C, and 1 min at 72°C; and a final step of 72°C for 1 min. The 12.5 µl PCR reaction mixes included 6.25 µl of 10% trehalose, 2.00 µl of ultrapure water, 1.25 µl 10× PCR buffer [200 mM Tris-HCl (pH 8.4), 500 mM KCl], 0.625 µl MgCl_2_ (50 mM), 0.125 µl of each primer [0.01 mM, using LCO1490/HCO2198 [28] with M13 tails], 0.062 µl of each dNTP (10 mM), 0.060 µl of Platinum® Taq Polymerase (Invitrogen), and 2.0 µl of DNA template. PCR amplicons were visualized on a 1.2% agarose gel E-Gel® (Invitrogen) and bidirectionally sequenced using sequencing primers M13F or M13R and the BigDye® Terminator v.3.1 Cycle Sequencing Kit (Applied Biosystems, Inc.) on an ABI 3730 capillary sequencer following manufacturer's instructions.

Sequences were edited in CHROMAS 2.3 (Technelysium, Queensland, Australia), and aligned using CLUSTAL [29] in MEGA v 5.0 [30]. Net sequence divergences among taxa were estimated in MEGA v 4.1 [28]. Maximum likelihood and Bayesian analyses were performed using a nucleotide substitution model selected in Modeltest version 0.1.1 [31] using BIC and AIC criteria, and the TIM+I+G model was selected for both analyses. COI sequences in GenBank for five northern hemisphere taxa: *R. inflata*, *R.* sp 28 California, *R. aculeata*, *R. inflata*, and *R. helleri* were included in phylogenetic analyses. Maximum likelihood analysis was done using PAUP v. 4b10 [32], with support for each internode evaluated by 1000 bootstrap replications [33]. Bayesian phylogenetic analyses were estimated with MrBayes version 3.1.2 [34]. Four simultaneous Monte Carlo chains were run for 1×10^6^ generations, saving the current tree every 1000 generations. Consensus trees with posterior probabilities were created with a burnin value equal to 1000 (the first 1000 trees were discarded). COI sequences for an Antarctic *Eusirus* species were used to root the trees. *Eusirus* is closely related to *Rhachotropis* and also belongs to the family Eusiridae. COI sequence data are available in BOLD and GenBank (Table 1).

### Morphological description

The specimen of the new species was dissected under a Leica MZ12 stereomicroscope and drawn using a camera lucida. All illustrations were digitally ‘inked’ following Coleman [35], [36]. Inking was done with the software Adobe Illustrator 14.0 and an A3 drawing table (Wacom Intuos 9×12).

Parts of selected specimens (mouthparts, antennae, coxal plates) were dried, coated with gold-paladium and investigated via a Scanning electron microscope LEO1525.

#### Nomenclatural Acts

The electronic version of this document does not represent a published work according to the International Code of Zoological Nomenclature (ICZN), and hence the nomenclatural acts contained in the electronic version are not available under that Code from the electronic edition. Therefore, a separate edition of this document was produced by a method that assures numerous identical and durable copies, and those copies were simultaneously obtainable (from the publication date noted on the first page of this article) for the purpose of providing a public and permanent scientific record, in accordance with Article 8.1 of the Code. The separate print-only edition is available on request from PLoS by sending a request to PLoS ONE, 1160 Battery Street, Suite 100, San Francisco, CA 94111, USA along with a check for $10 (to cover printing and postage) payable to “Public Library of Science”.

In addition, this published work and the nomenclatural acts it contains have been registered in ZooBank, the proposed online registration system for the ICZN. The ZooBank LSIDs (Life Science Identifiers) can be resolved and the associated information viewed through any standard web browser by appending the LSID to the prefix “http://zoobank.org/”. The LSID for this publication is:

urn:lsid:zoobank.org:pub:B21B0DED-2543-40F0-BB02-3883DF06A245

The LSID for *Rhachotropis novazealandica* spec. nov. is:

urn:lsid:zoobank.org:act:F270B26E-A63D-42A2-B9F0-62A502E2EFB4

## References

[1] Lörz A-N (2010). Deep-sea *Rhachotropis* (Crustacea: Amphipoda: Eusiridae) from New Zealand and the Ross Sea with key to the Pacific, Indian Ocean and Antarctic species.. Zootaxa.

[2] d'Udekem d'Acoz C, Vader W, Legezinska J (2007). On a new diminutive *Rhachotropis* species from the North Sea, with a key to European *Rhachotropis* (Crustacea, Amphipoda, Eusiridae).. Boll Mus Civ Stor Nat Verona Bot Zool.

[3] Cartes JE, Sorbe JC (1999). Deep-water amphipods from the Catalan Sea slope (western Mediterranean): Bathymetric distribution, assemblage composition and biological characteristics.. J Nat Hist.

[4] Bousfield EL, Poinar GO (1995). New terrestrial amphipod from tertiary amber deposits of the Dominican Republic.. J Crust Biol.

[5] Ratnasingham S, Hebert PDN (2007). BOLD: The Barcode of Life Data System (www.barcodinglife.org).. Mol Ecol Notes.

[6] Costa FO, deWaard JR, Boutillier J, Ratnasingham S, Dooh RT (2007). Biological identifications through DNA barcodes: the case of the Crustacea.. Can J Fish Aquat Sci.

[7] Ward RD, Hanner R, Hebert PDN (2009). The campaign to DNA barcode all fishes, FISH-BOL.. J Fish Biol.

[8] Hebert PDN, Cywinska A, Ball SL, DeWaard JR (2003). Biological identifications through DNA barcodes.. Proceedings of the Royal Society of London Series B-Biological Sciences.

[9] Bucklin A, Steinke D, Blanco-Bercial L, Carlson CA, Giovannoni SJ (2011). DNA Barcoding of Marine Metazoa.. Annual Review of Marine Science, Vol 3.

[10] Lörz A-N, Linse K, Smith PJ, Steinke D (2012). High genetic diversity within *Epimeria georgiana* (Amphipoda) from the southern Scotia Arc.. Mar Biodiv.

[11] Carreon-Martinez L, Johnson TB, Ludsin SA, Heath DD (2011). Utilization of stomach content DNA to determine diet diversity in piscivorous fishes.. J Fish Biol.

[12] Dunn MR, Szabo A, McVeagh MS, Smith PJ (2010). The diet of deepwater sharks and the benefits of using DNA identification of prey.. Deep-Sea Research Part I-Oceanographic Research Papers.

[13] Smith PJ, McVeagh SM, Steinke D (2008). DNA barcoding for the identification of smoked fish products.. J Fish Biol.

[14] Wong EHK, Hanner RH (2008). DNA barcoding detects market substitution in North American seafood.. Food Res Int.

[15] Hajibabaei M, Shokralla S, Zhou X, Singer GAC, Baird DJ (2011). Environmental Barcoding: A Next-Generation Sequencing Approach for Biomonitoring Applications Using River Benthos.. PLoS ONE.

[16] Vogler AP, Monaghan MT (2007). Recent advances in DNA taxonomy.. J Zool Syst Evol Res.

[17] Held C, Leese F (2007). The utility of fast evolving molecular markers for studying speciation in the Antarctic benthos.. Polar Biol.

[18] Lörz A-N, Maas EW, Linse K, Coleman CO (2009). Do circum-Antarctic species exist in peracarid Amphipoda? A case study in the genus *Epimeria* Costa, 1851 (Crustacea, Peracarida, Epimeriidae).. ZooKeys.

[19] Lörz AN, Held C (2004). A preliminary molecular and morphological phylogeny of the Antarctic Epimeriidae and Iphimediidae (Crustacea, Amphipoda).. Mol Phylogen Evol.

[20] Lowry JK, Springthorpe RT (2005). New calliopiid and eusirid amphipods from eastern Australian waters (Crustacea : Amphipoda : Calliopiidae : Eusiridae).. Proc Biol Soc Wash.

[21] Bellan-Santini D (2006). Rhachotropis species (Crustacea : Amphipoda : Eusiridae) of hydrothermal vents and surroundings on the Mid-Atlantic Ridge, Azores Triple Junction zone.. J Nat Hist.

[22] Griffiths HJ, Danis B, Clarke A (2011). Quantifying Antarctic marine biodiversity: The SCAR-MarBIN data portal.. Deep Sea Res (II Top. Stud. Oceanogr.).

[23] Brandt A, Linse K, Schuller M (2009). Bathymetric distribution patterns of Southern Ocean macrofaunal taxa: Bivalvia, Gastropoda, Isopoda and Polychaeta.. Deep Sea Res (I Oceanogr Res Pap).

[24] Hessler RR, Thistle D (1975). On the place of origin of deep-sea isopods.. Mar Biol.

[25] Jamieson AJ, Fujii T, Mayor DJ, Solan M, Priede IG (2010). Hadal trenches: the ecology of the deepest places on Earth.. Trends Ecol Evol.

[26] Whitworth T, Warren BA, Nowlin WD, Rutz SB, Pillsbury RD (1999). On the deep western-boundary current in the Southwest Pacific Basin.. Prog Oceanogr.

[27] Ivanova NV, Dewaard JR, Hebert PDN (2006). An inexpensive, automation-friendly protocol for recovering high-quality DNA.. Mol Ecol Notes.

[28] Folmer O, Black M, Hoeh W, Lutz R, Vrijenhoek R (1994). DNA primers for amplification of mitochondria; cytochrome c oxidase subunit I from diverse metazoan invertebrates.. Mol Mar Biol Biotech.

[29] Thompson JD, Higgins DG, Gibson TJ (1994). “CLUSTAL W: improving the sensitivity of progressive multiple sequence alignment through sequence weighting, position-specific gap penalties and weight matrix choice.”. Nucleic Acids Res.

[30] Tamura K, Dudley J, Nei M, Kumar S (2007). MEGA4: Molecular evolutionary genetics analysis (MEGA) software version 4.0.. Mol Biol Evol.

[31] Posada D (2008). jModelTest: Phylogenetic model averaging.. Mol Biol Evol.

[32] Swofford DL (2003). PAUP*: phylogenetic analysis using parsimony (*and other methods). Version 4.

[33] Felsenstein J (1985). Confidence limits on phylogenies: an approach using the bootstrap.. Evolution.

[34] Ronquist F, Huelsenbeck JP (2003). MrBayes 3: Bayesian phylogenetic inference under mixed models.. Bioinformatics.

[35] Coleman CO (2003). “Digital inking”: how to make perfect line drawings on computers.. Org Divers Evol.

[36] Coleman CO (2009). Drawing setae the digital way.. Zoosyst evol.

